# Dual Optical Nanosensor Based on Ormosil Nanoparticles for Monitoring O_2_ and pH

**DOI:** 10.3390/bios12111011

**Published:** 2022-11-12

**Authors:** Reham Ali

**Affiliations:** 1 Department of Chemistry, College of Science, Qassim University, Buraidah 51452, Saudi Arabia; re.ali@qu.edu.sa; 2 Chemistry Department, Science College, Suez University, 43518 Suez, Egypt

**Keywords:** pH sensor, pO_2_ sensor, ormosil, salicylamide, fluorescent nanosensor, dual sensor

## Abstract

Monitoring O_2_ and pH has excellent potential in different sensing applications, especially in biological and clinical applications. This report presents a protocol for synthesizing an optical dual nanosensor for those two parameters. The organically modified silica (ormosil) nanoparticles were prepared based on phenytrimethoxysilane in an aqueous solution using an acid-base one-pot strategy. Ormosil was selected as a lipophilic matrix for loading fluorescent O_2_-sensitive dye platinum(II)-tetrakis-(pentafluorophenyl) porphyrin (Pt-TPFPP), which was quenched in the presence of O_2_ gas and exhibited a considerable detection proficiency within a percentage range of (0–100%) O_2_. Commercially available drug ingredient salicylamide was labeled on the surface of the nanoparticles using a coupling agent (3-glycidoxypropyl) trimethoxysilane (GPTMS). For measuring pH, salicylamide acted for the first time as a pH-sensitive probe based on a turn-on process with increasing pH. The nanosensor displayed a significant pH detection efficiency in the range of (pH = 6–10). Salicylamide turn-on fluorescence was attributed to the excited state intramolecular transfer (ESIPT) process followed by the inter charge transfer (ICT). The presented dual nanosensor opens new opportunities as a promising candidate material for industrial systems and medical applications.

## 1. Introduction

With the spread of chemical sensors in many fields, there was an urgent need to develop multi-functional nanosensors. This type of sensor requires new materials with some specifications and standards. The essential standards of these materials are to be compatible, safe, and non-toxic, especially for medical applications. Therefore, scientists have recently tried to design new materials for chemical sensing. pH and pO_2_ are chemical parameters of significant interest in industrial, environmental, and medical applications. They are needed to be measured together and monitored continuously with high accuracy in different applications, especially in the medical and biochemical fields [1,2]. They can help the understanding of cellular behavior.

Optical sensors are a promising tool for monitoring those two parameters. Fluorescence has high-significance advantages in terms of the precision and accuracy of optical sensors [3,4,5]. Different probes have been used for this purpose, including organic fluorophores and polymers [6,7,8,9]. However, most are not biocompatible and only based on measuring one parameter. Fluorescence sensors, including measuring two parameters simultaneously, are rare. They save time, effort, and cost [10]. We can achieve this feature by applying two standard fluorophores that can be excited at the same wavelength to obtain two different signals at two different wavelengths to work as a dual sensor. However, the challenge is to find a suitable matrix for both fluorophores, as gas sensors need hydrophobic materials with high permeability, while sensors for pH require hydrophilic materials.

Fluorescent silica nanoparticles are still one of the most preferred chemical and biochemical sensing materials. Silica nanoparticles have no fluorescence but can be doped or immobilized with other fluorophores on their surface [11]. They have high stability, biocompatibility, and non-toxicity. Silica nanoparticles can be prepared using sol-gel technology through the hydrolysis and condensation of suitable inorganic metal alkoxides as glass-like or ceramic materials are produced [12,13]. Silica nanoparticles have been recently reported for detecting several analytes, such as pH [14]. However, this type of silica has low porosity and is unsuitable for doping with organic molecules for sensing gases. Using alternative inorganic precursors such as alkyl-substituted silicon alkoxide produces better properties than inorganic silane nanoparticles. Introducing the organic groups to the network of silicon increases the hydrophobicity and porosity of the nanoparticles. These modified materials are called organically modified silica (ormosil) nanoparticles. These nanoparticles can be prepared at low temperatures, and their compositions change according to the organic precursors. They possess many advantages, including their ability to act as a suitable host for different organic dyes by incorporating fluorescent active dyes into them [15]. Furthermore, these nanoparticles are water-dispersible, making them a biocompatible matrix for carrying hydrophobic dyes in aqueous media that open the way for different applications in the chemical and medical industries [16]. 

Ormosil nanoparticles display a high porosity and surface area compared to the silicone-based sensor, which allow higher concentrations of reagents to be incorporated into the matrix, resulting in improved sensor sensitivity. Additionally, they enhance the photochemical properties, including absorption and emission. Moreover, several types of reactive functional groups can be introduced in their matrix, which can be successively used for specific covalent labeling of other molecular recognition groups [17,18]. Ormosil is a promising candidate for all these advantages as a matrix nanosensor for an efficient and suitable use for sensing gases and pH. 

Salicylamide is a commercially available hydrophilic material. It is the common name for the substance o-hydroxybenzamide. It has an analgesic and antipyretic activity [19]. Salicylamide is used combined with other drugs such as acetaminophen, aspirin, and caffeine to treat migraine and pain in addition to significant cytotoxic activity [20]. Few reports study the optical properties of salicylamide. Salicylamide has never been used as a fluorophore in optical sensors. However, it is highly sensitive to measuring the pH of the solution-based ESIPT (excited state intramolecular proton transfer) mechanism [21,22]. However, it is insensitive to O_2_.

A lipophilic oxygen-sensitive dye, platinum porphyrin, has been widely used for pO_2_ optical sensors [23,24,25]. However, the hydrophobicity of the probe limited its biological applications. Platinum porphyrin doped in ormosil coating was used as an oxygen sensor exhibiting high oxygen sensitivity and quantum yield [26]. Platinum porphyrin dyes have a long excitation-emission wavelength with significant Stokes shifts and high quantum yields, which result in reduced background light scattering and autofluorescence [27].

Various dual fluorescent chemical sensors for pH and pO_2_ have been reported. Most are designed as planer hydrogel [28] or polymer films [29,30,31,32]. Dual fluorescent nanosensors allow both intracellular and extracellular applications [33]. They are established on the immobilization of two probes on the surface of the nanoparticles. Other sensors are based on the immobilization of one probe on the surface and the other doped inside the nanoparticles, provided that the analyte can penetrate the particles and reach the indicator dye, like gasses.

Here, we introduce a novel strategy for introducing nanosensors with single excitation-dual emission fluorescence for pO_2_ and pH. The pH probe salicylamide has never been used previously for optical sensing. The ormosil nanosensor was synthesized using acid-base hydrolysis in the one-pot method. They were doped with sensitive O_2_ probe platinum(II)-tetrakis-(pentafluorophenyl) porphyrin (Pt-TPFPP), and then the surface was modified with an epoxy group which enabled labeling of pH probe salicylamide.

## 2. Experimental

### 2.1. Reagents and Materials

The 3-Glycidoxypropyl)trimethoxysilane (GPTMS) and Phenyltrimethoxysilane (PTMS) were purchased from Alfa Aesar. (Lancashire, England). The O_2_ sensitive probe Pt (II) 5,10,15,20-tetrakis(2,3,4,5,6-pentafluorophenyl)-porphyrin (Pt-TPFPP) was purchased from Porphyrin Systems. All other chemicals were, in this work, of the analytical-reagent grade and used without further purification. For the pH calibration of the nanosensor, we prepared different pH solutions ranging from 2 to 12 using a stock solution of 20 mM Britton Robinson (BR) universal buffer. Then, the different solutions with pH values were adjusted using appropriate volumes of 1 M HCl or NaOH solution. Dry nitrogen and oxygen gases (99.99% purity) were used to obtain standard gas mixtures with different percentages of oxygen ranging from 0 to 100% using gas flowmeters. The gas mixtures were passed through the nanosensor sample’s cuvette to record the nanosensor’s luminescent emission spectra at atmospheric pressure at room temperature. Response times and reversibility of the O_2_ nanosensor were determined by detecting the time traces of the emission peak at 648 nm.

### 2.2. Instruments

The absorption spectra (UV–Vis) were recorded using Evolution™ 200 series (Thermo Fisher Scientific, Waltham, MA, USA) UV–Visible spectrophotometer. Fluorescence spectra of the probes and nanosensor were measured with JASCO FP6300 spectrofluorometric using a quartz luminescence-free cell with a path length of 1 cm and 5 nm bandpass excitation and emission filters. Transmission electron microscopy images of ormosil nanosensors were recorded using a JEOL-100S, Japan. Dynamic light scattering (DLS) analyses of the synthesized ormosil nanoparticles were recorded.

Using Malvern Instrument (Malvern, UK), the Fourier transform infrared spectroscopies (FT-IR) were measured using a Perkin Elmer FT-IR spectrometer. A thermogravimetric analysis (TGA) was measured using a Perkin Elmer thermal analyzer at a heating rate of 10 °C min^−1^ under nitrogen.

### 2.3. One-Pot Synthesis of Ormosil Nanosensor

A 50 mL round-bottomed flask was washed with ethanol and ultrapure water and allowed to air dry. Next, 31 mL of deionized water was added to the round flask, and the temperature was adjusted in a water bath at 60 °C. Then, 38 µL of nitric acid HNO_3_ 25% was added to the flask, and the solution kept stirred. Then, 100 µL of PTMS was added to the reaction. After 20 min, 6 mL of ammonium hydroxide was added, and the reaction proceeded for two hours as the solution became milky [34]. Next, 1.5 mg of the oxygen-sensitive dye PtTPFPP soluble in 1 mL of THF was added to the reaction mixture, which was kept stirring for an additional hour, and then 25 µL of GPTMS was added into the mixture [35]. After 24 h, 127 µL (1 mg/mL) of salicylamide was added, and the reaction proceeded for another three hours. The resulting ormosil nanoparticles were centrifuged at 4000 rpm, rinsed three times with water, and resuspended in a water/ethanol 1:2 mixture with sonication for 5 min, then centrifuged again and allowed to air-dry. The obtained nanosensor was characterized using different methods.

### 2.4. UV-Vis and Fluorescence Measurements for Salicylamide and Ormosil Nanosensor

The UV-Vis and fluorescence spectra of salicylamide were measured in an aqueous solution of 20 mM BR buffer at different pH ranges from 2 to 12. For all measurements, the excitation and emission slit widths were 5 nm. To investigate the response of the ormosil nanosensor, 0.4 mg/mL of the nanosensor was utilized in an aqueous BR buffer solution (20 mM) for fluorescence titration. For pH calibration, the intensity of the emission spectra at 457 nm was recorded after adjusting the pH of the suspended nanosensor inside the cuvette within the range of the buffer solution of different pH values. Oxygen calibrations were performed using a buffer of pH 7 bubbled with standard O_2_/N_2_ gas mixtures (0–100% O_2_). Upon gas equilibration, the intensity at 648 nm was measured at room temperature in atmospheric pressure.

## 3. Results

### 3.1. Choice of Materials and Method of Preparation

The dual nanosensor was prepared based on the ormosil matrix. PTMS was chosen as a precursor for building the ormosil nanoparticles using the acid-base hydrolysis method. Ormosil-based PTMS behaves as a suitable hydrophobic host material for the O_2_ probe Pt-TPFPP in the core and allows the immobilization of the pH probe salicylamide on the surface. Ormosil is proper for doping the O_2_ probe due to the organic moiety inside the nanoparticles and provides high permeability for sensing O_2_ gas. Ormosil has many advantages over other sensing matrixes materials such as being optically transparent, high chemical stability, bioinert material, and that it can be loaded with hydrophobic and hydrophilic molecules [36]. 

Although the Pt-TPFPP probe has been widely reported for sensing oxygen gas, most of the reported materials doped with Pt-TPFPP as a dual optical sensor for pH and O_2_ are primarily based on planer thin films or polymer nanosensors [26,37]. Salicylamide is a water-soluble, inexpensive, and commercial material. Different studies have been reported on its analgesic and antipyretic properties. It has been used as a drug component for treating migraine and pain. Salicylamide has never been reported as an optical pH probe, although it exhibits an intense fluorescence turn-on in a basic medium. So, we present here a new fluorescent pH-sensitive probe. At the same time, it can be easily immobilized to the surface of the Pt-PTFPP-doped ormosil nanoparticles via GPTMS as a linker in one-pot synthesis rot. To obtain the target, the epoxy-modified ormosil nanoparticles were reacted with the amide group of salicylamide in solution in a basic aqueous medium for immobilization. Worth noting, the two probes mentioned above can be excited at the same wavelength (365 nm), simultaneously providing dual sensing for the pH and O_2_. 

**Scheme 1:**
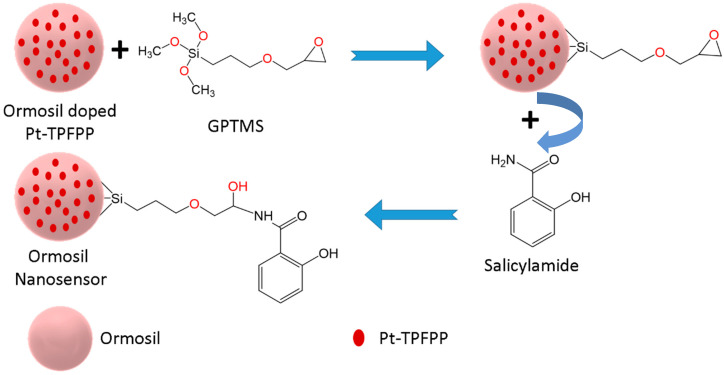
Schematic for synthesizing ormosil nanosensors dopped with O_2_ probe Pt-TPFPP and immobilized with pH probe salicylamide.

The preparation reaction of ormosil proceeded in an aqueous solution based on the acid-base sol-gel process. Where the acid allowed the hydrolysis of the precursor PTMS, a milky solution was formed. This was followed by the alkylation of the solution using ammonium hydroxide, as the co-condensation started to occur, and the reaction was continued. Then, the dopant O_2_ probe Pt-TPFPP was introduced to the reaction. The hydrophobic host moiety of the nanoparticles facilitated the entrapment of Pt-TPFPP, eliminating leaching effects and obtaining a homogeneous solution of the Pt-TPFPP-doped ormosil nanoparticles. The linker GPTMS was added as it has three methoxy groups hydrolyzed in water. Condensation occurred between GPTMS and the ormosil nanoparticles, using an essential catalyst as the reaction rate of condensation increased [35]. The schematic mechanism of the reaction is shown in Scheme 1. The synthesized nanosensor showed excellent stability and dispersibility in water at a different pH, and no precipitation was observed in the solution.

### 3.2. Structural Characterization of the Ormosil Nanosensor

**Figure 1:**
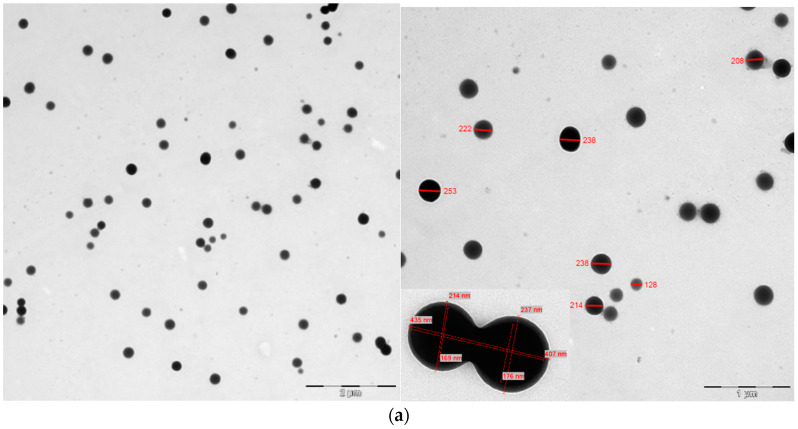

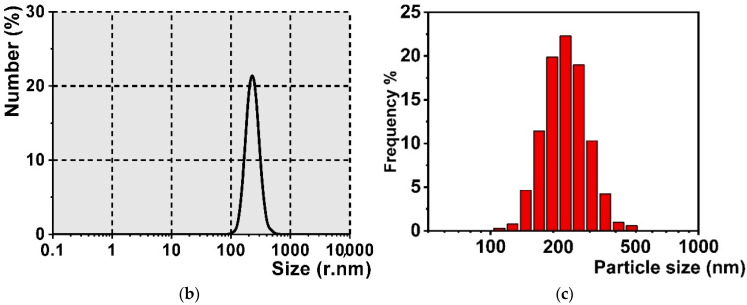
(**a**) TEM images; (**b**) DLS; (**c**) particle size distribution of the as-prepared ormosil nanosensor.

Characterization of the nanosensor was managed using different methods, including FTIR, TGA, TEM, and DLS. TEM images of the ormosil nanoparticles are shown in Figure 1a. The synthesized nanoparticles’ size was assigned to be 227 nm. Furthermore, the dynamic light scattering DLS measurements and particle size distribution approved the nanoparticles’ approximate size (see Figure 1b and Figure 1c, respectively). The DLS data showed that the mean size was 230 nm with std 3.33 for the synthesized nanoparticles.

**Figure 2:**
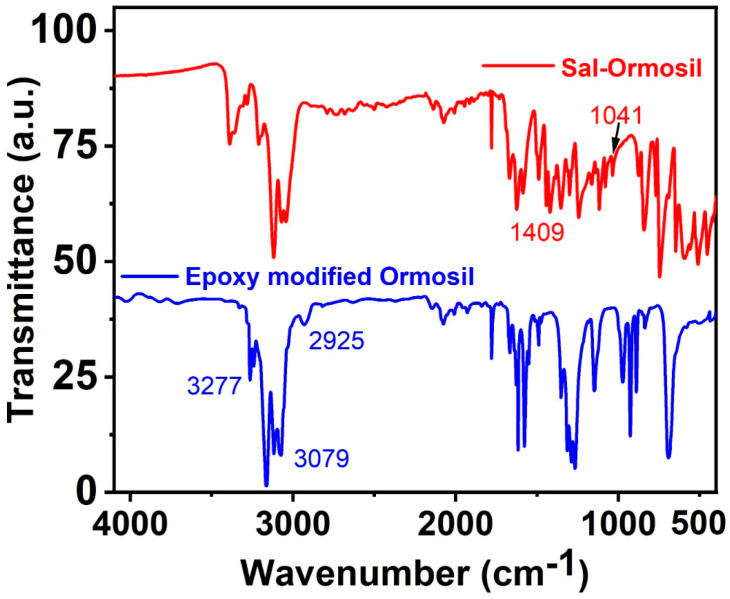
FT-IR of the epoxy-modified ormosil (**blue**) and the covalent immobilization of salicylamide to the ormosil nanoparticles (**red**).

The IR-Analysis is an essential characteristic technique for the most straightforward proficient detection of multiple functional groups of nanoparticles [38]. FT-IR spectra were used to confirm the ormosil nanoparticles’ structure. Figure 2 shows the FT-IR analysis of epoxy-modified ormosil and salicylamide immobilization ormosil nanoparticles after modification. The spectra of epoxy-modified ormosil showed the characteristic band at 3277 cm^−1^ attributed to stretching N–H of core porphyrin, and significant bands of aromatic (3079 cm^−1^) and aliphatic (2925 cm^−1^) v(C-H) that can be ascribed to the stretching vibrational bands from phenyl and epoxy groups, correspondingly [17]. Further, the FT-IR spectrum of salicylamide-immobilized ormosil nanoparticles was measured. This recognized the numerous peaks in the functional group district peaks area of the salicylamide-labeled particles spectrum (1409 cm^−1^) and the second hydroxyl absorbance peak delocalized at 1379 and 1041 cm^−1^,respectively [39]. Further, the prominent absorption peak of hydroxyl and amine groups occurred around 3400 cm^−1^. Finally, the disappearance of the epoxy peak at 2925 cm^−1^ proved the surface labeling of the nanoparticles with salicylamide molecules.

**Figure 3:**
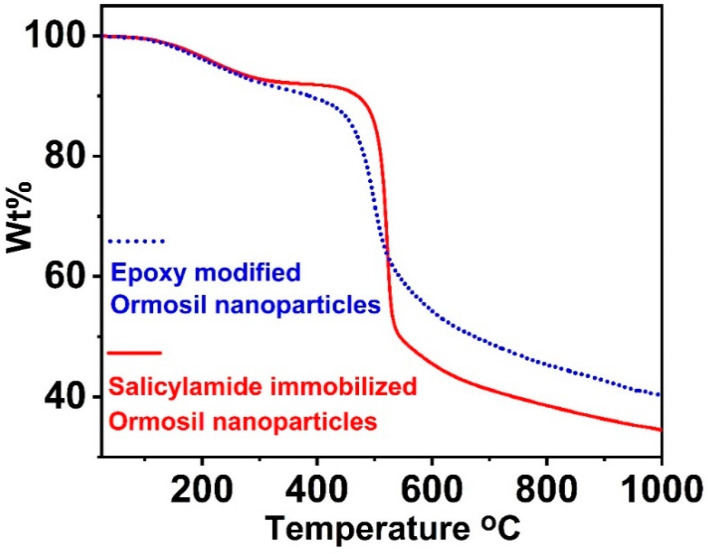
TGA of the salicylamide-immobilized ormosil nanoparticles before (**blue**) and after (**red**) immobilization.

A thermogravimetric analysis (TGA) for the epoxy-modified ormosil nanoparticles before and after immobilization with salicylamide was utilized to investigate the surface modification. TGA characterized the composition of the two synthesized nanomaterials (Figure 3). The samples’ treatment with oxygen or air escorted to intense exothermic reactions and irreversible TG diagrams. Both samples exhibited a minor mass loss within a temperature range of 100–400 °C; this could be attributed to the thermal degradation of the solvent and water molecules. These degradable molecules were bonded to the nanoparticles’ surface based on the hydroxyl groups by condensation reaction. The weight loss was continued due to the thermal degradation of the organic molecules joined to Si atoms. Salicylamide-immobilized ormosil nanoparticles had greater thermal temperatures and weight loss than epoxy-modified ormosil. The results exhibited a difference in the weight loss between the nanoparticle before and after the immobilization of salicylamide. Thus, data proved that the salicylamide molecules were successfully attached to the surface of the nanoparticles.

### 3.3. Optical Properties of Salicylamide and the Ormosil Nanosensor

**Figure 4:**
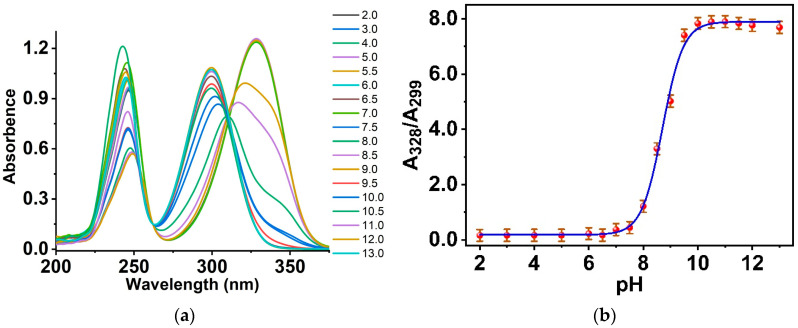
(**a**) Absorption spectra of salicylamide in aqueous solution at different pH values; (**b**) calibration curve of the absorption spectra of salicylamide.

The absorption spectra of salicylamide in an aqueous solution at different pH values are shown in Figure 4a. The spectra in the neutral solution showed two prominent peaks centered at 240 and 303 nm. The absorption bands were assigned to π-π* and n-π* transitions. The pk_a_ of salicylamide was recorded in an aqueous solution using a range of buffer solutions of different pH values and assigned to 8.1. With an increase in the pH from the acidic to neutral medium, a redshift has occurred in the absorbance peak at 299, and a new peak has appeared at 328 nm. When the pH increased to the basic, an increase in the absorbance of the peak located at 328 nm synchronized with a decrease in the absorbance maxima at the 299 nm peak. The calibration curve of the absorption peaks’ ratio (A_328_/A_299_) of the salicylamide versus pH of the studied solutions is exhibited in Figure 4b.

**Figure 5:**
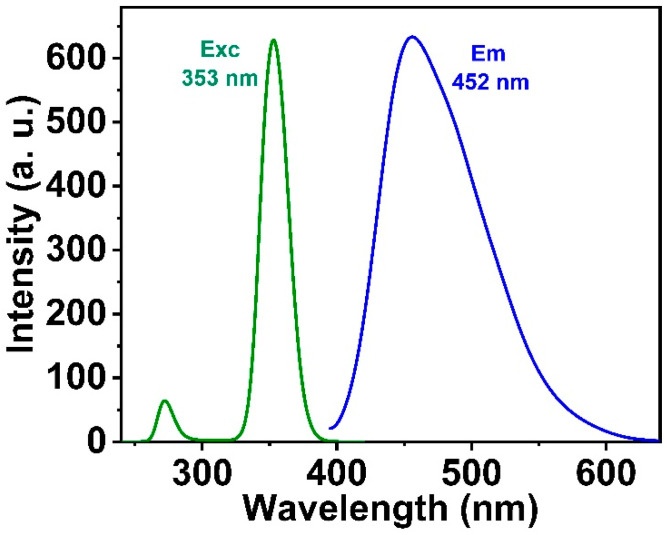
Excitation and emission spectra of salicylamide in aqueous solution.

Salicylamide has an emission band centered at 452 nm in an aqueous solution under excitation at 353 nm, as shown in Figure 5, with a significant Stokes shift of approximately 100 nm. Very little interest has been paid to studying the fluorescence properties of salicylamide [22,40].

**Figure 6:**
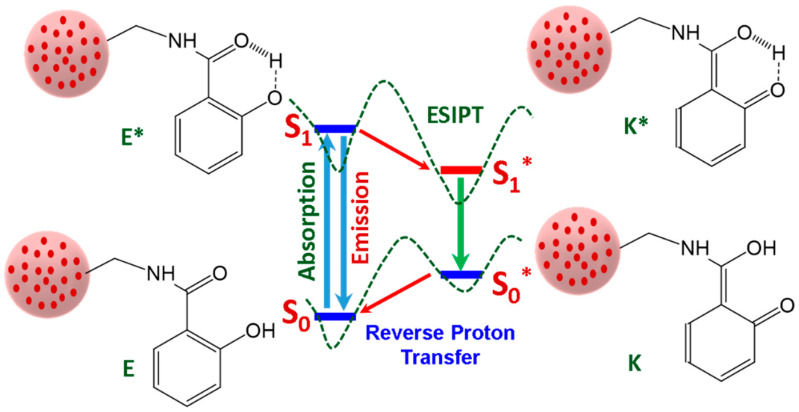
The schematic diagram for the intramolecular proton transfer (ESIPT) mechanism in salicylamide-immobilized ormosil nanosensor; E and K are the enol and keto tautomer species, respectively. The asterisk sign indicates the excited state.

The emission spectra of salicylamide were based on the excited state intramolecular proton transfer (ESIPT) process, in which the excitation of the salicylamide molecule produces a photo-induced proton tautomerization from enol form (E*) to the keto form (K*), associated with proton migration from the hydroxyl group to the oxygen atom of the carbonyl group (-C=O). The enol form was recovered by reverse proton transfer after the relaxation of the salicylamide’s keto form (see Figure 6). The emission from K* fluorescence was produced with a significant Stokes shift; however, low or non-emission from E* occurred. This process is affected by different parameters and is sensitive to the pH and the solvent of the surrounding medium. This is attributed to the influence of the solvent on the intramolecular H-bonding between a proton donor (–OH) and a proton acceptor (C=O) of the probe.

**Figure 7:**
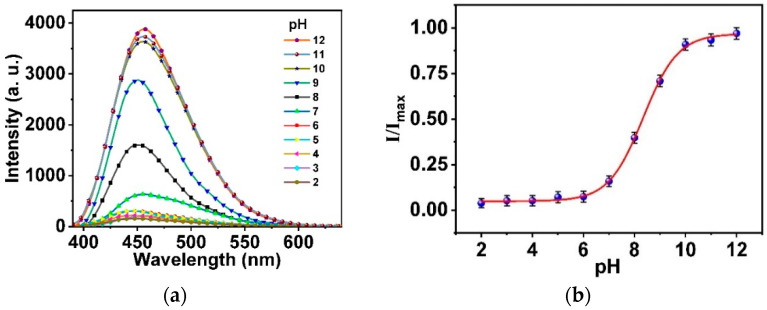
(**a**) Emission spectra of salicylamide in aqueous solution at different pH ranging (2–12) upon excitation with 350 nm; (**b**) calibration curve of I/I_max_ (452 nm) at different pH.

To understand the fluorescence properties of salicylamide, the emission spectra were measured at different pH. In the acidic and neutral medium, salicylamide was present in a protonated form with an intramolecular hydrogen bond between (OH) and (C=O), which provided the accessibility of the ESIPT process ascribed to the tautomeric equilibrium. Meanwhile, a substantial fluorescence intensity enhancement at higher pH is attributable to the internal charge transfer (ICT) process [41]. With the increased pH of the medium, salicylamide started to deprotonate with no intramolecular hydrogen bonding. However, a donor-acceptor charge transfer occurred, and an intense turn-on was observed in the emission with a small redshift. The charge transfer mechanism was performed from the electron-rich phenolate anion to the electron-deficient benzene ring. As a result, a negative charge was delocalized over the conjugated system. Salicylamide had a weak fluorescence in the acidic medium. An intense turn-on of about 83% was observed in the intensity from the neutral to the basic medium (pH 12), as shown in Figure 7a. There was a calibration curve of I/I_max_ at 452 nm of salicylamide in aqueous solution 20 mM BR buffer versus pH range of (2–12) (see Figure 7b). A substantial enhancement of the emission peak of salicylamide was observed by increasing the pH value from 6 to 12. Further, a slight redshift ~5nm in the emission peak at pH 10 to 12 was observed. For comparison, salicylamide was studied in different oxygen concentrations upon excitation with 365 nm. The peak at 457 nm had no change in the presence of different O_2_ concentrations, indicating the insensitivity of the salicylamide to O_2_ concentration.

**Figure 8:**
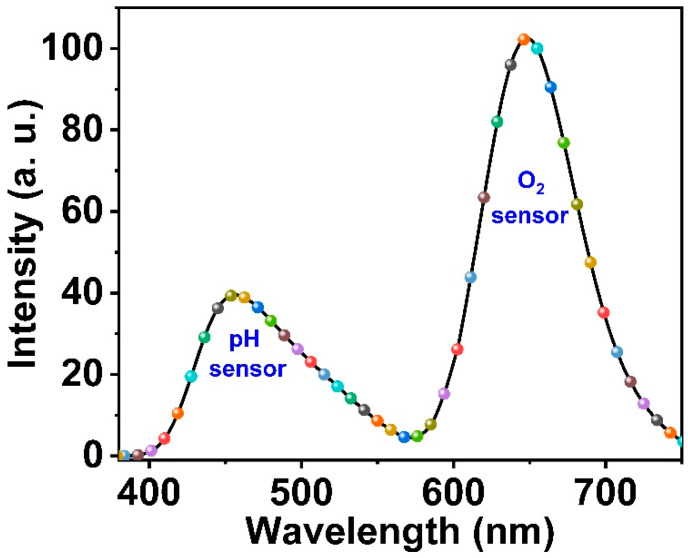
The emission spectrum of the ormosil nanosensor doped with Pt-TPFPP and immobilized with salicylamide under excitation with 365 nm.

The synthesized ormosil nanosensor doped with Pt-TPFPP and immobilized with salicylamide had an excitation wavelength (365 nm) as the emission peak of salicylamide detected at 457 nm with a minimal shift in the maximum peak ~5 nm. This can be attributed to labeling salicylamide molecules to the ormosil nanoparticles. The emission of Pt-TPFPP was observed at 648 nm, as shown in Figure 8.

**Figure 9:**
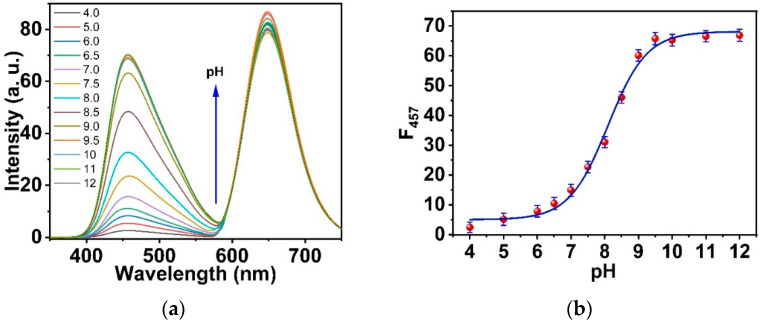
(**a**) The emission of the ormosil nanosensor in different pH buffer solutions ranging from 4–12; (**b**) the corresponding calibration curve of the intensity at 457 nm of the ormosil nanosensor at different pH.

Moreover, Pt-TPFPP doped in the ormosil nanoparticles was insensitive to pH changes. Upon excitation at 365 nm, the nanosensor emitted blue and red fluorescence at 457 nm and 648 nm respectively. The blue emission at 457 nm has pH-dependent fluorescence however, the red emission at 648 nm is pH-insensitive. The titrimetric reaction of the optical nanosensor with a changing pH of the solution within the range of (4 to 12) using 20 mM RB buffer is exhibited in Figure 9a. The calibration curve intensity at 457 nm versus the pH of the solution is shown in Figure 9b.

**Figure 10:**
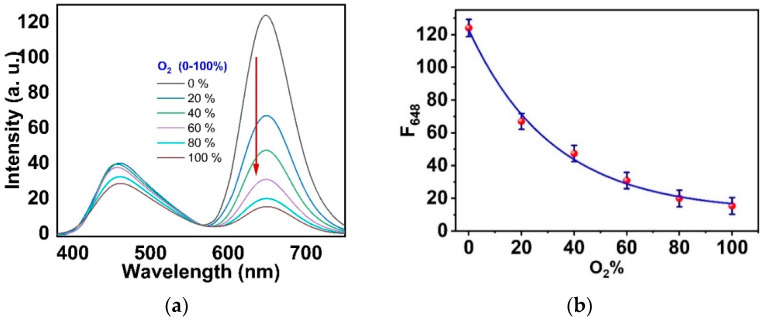
(**a**) The emission of the ormosil nanosensor at different concentrations of O_2_ (0–100%); (**b**) the calibration curve of the nanosensor at different oxygen concentrations upon excitation with 365 nm.

Additionally, we examined the ormosil nanoparticles as an O_2_ nanosensor, which showed high performance. The titrimetric reaction of the nanosensor using different concentrations of oxygen gas is demonstrated in Figure 10a. The excess oxygen concentration provided a significant quenching of the Pt-TPFPP dye. On the other hand, salicylamide was insensitive to O_2_. To investigate the sensitive performance of the oxygen sensor, we plotted the intensity of the red emission peak (at 648 nm for Pt-TPFPP) under excitation at 365 nm with oxygen concentrations of 0–100%, as shown in Figure 10b.

**Figure 11:**
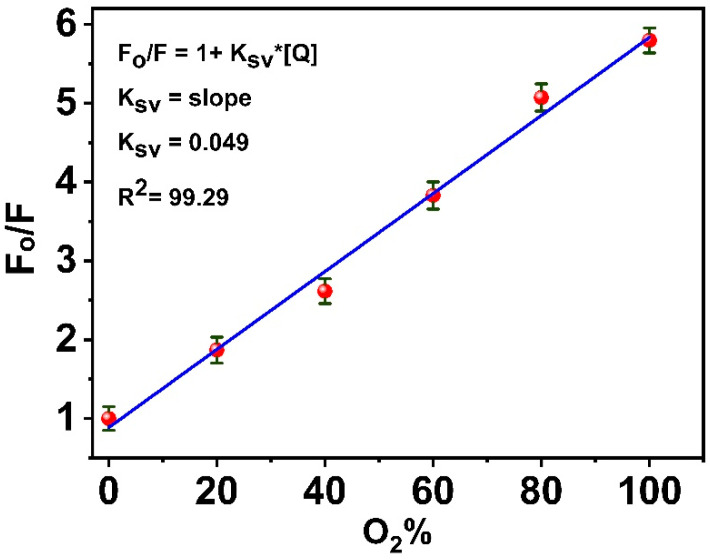
Stern–Volmer plot for the oxygen nanosensor for oxygen concentration in the 0–100% range.

The Stern–Volmer plot for the oxygen sensing is shown in Figure 11. The plot is linear, which indicates that the sensor was sensitive to oxygen in this range where the K_SV_ was 0.049%^−1^ and R^2^ was 99.29. The oxygen sensitivities (F_0_/F_100_) of the nanosensor were calculated to be about 6, where F_0_ and F_100_ were the luminescence intensities at 0 and 100% oxygen, respectively.

**Figure 12:**
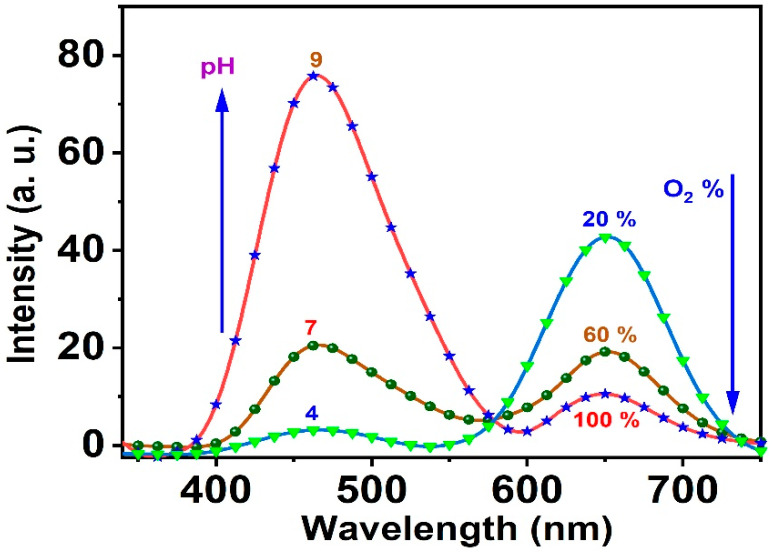
The emission of the ormosil nanosensor at different concentrations of O_2_ and pH values under excitation with 365 nm.

The response of the optical nanosensor was examined randomly at different pH values and oxygen percentages, and the conditions were chosen randomly to be conditions (1) pH 9, O_2_ 100%; conditions (2) pH 7, O_2_ 60%; and conditions (3) pH 4, O_2_ 20%. The system response was efficient and dealt with the previous preliminary data introduced at different pH values or oxygen percentages (see Figure 12).

### 3.4. Response, Response Time, and Reversibility of the Ormosil Nanosensor

**Figure 13:**
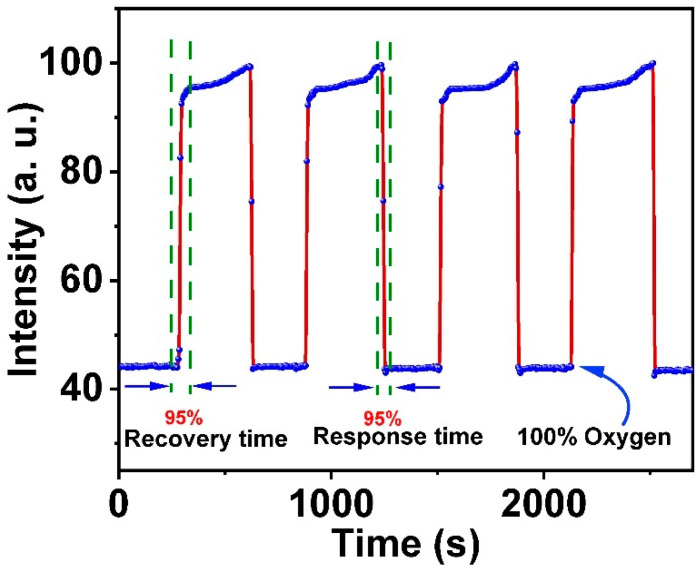
Response times and reversibility of the O_2_ ormosil nanosensor on switching between 100% O_2_ and 100% N_2_ under excitation at 365 nm; emission was detected at 648 nm.

The response time and reversibility of the nanosensor for O_2_ detection were studied by recording the intensity signals of the nanosensor at 648 nm on cycling between N_2_ and 100% of the saturated dissolved O_2_ (Figure 13). The response times (defined as the time for 95% of the signal change to occur) were ~30 s for switching from N_2_ to O_2_ and ~60 s for switching from O_2_ to N_2_. The signal changes were fully reversible. Furthermore, the pH nanosensor had a rapid equilibrium rate because the pH probe salicylamide was located at the surface of the nanosensor and exhibited good contact with the aqueous samples and complete reversibility.

## 4. Conclusions

Here, we present a dual fluorescent nanosensor for detecting two of the potential parameters in medical and industrial applications: O_2_ and pH. The nanosensor was based on ormosil nanoparticles produced from the organic precursor PTMS in the one-pot method. Ormosil nanoparticles were used as a proper matrix for hosting the two probes of O_2_ (Pt-TPFPP) and pH (salicylamide). Firstly, the nanoparticles were doped with the O_2_-sensitive probe Pt-TPFPP, and then modified with the linker GPTMS on the surface to link the second probe salicylamide on the surface for pH sensing. The nanosensor had a diameter of 227 nm. The surface modification was characterized using different methods, including FT-IR and TGA, to verify the existence of salicylamide on the ormosil surface. Upon excitation with 365 nm, blue and red fluorescence emitted at 457 and 648 nm corresponded to the presence of the two probes, salicylamide and Pt-TPFPP, respectively. The two emission peaks allowed a dual analysis for the two mentioned parameters, pH and O_2_. The nanosensor can detect the pH in the range of 6–10 and O_2_ in the 0–100% range. 

## Data Availability

Not applicable.
